# Muscle invasive urinary bladder urothelial carcinoma presenting with secondary nephrotic symptoms

**DOI:** 10.1002/iju5.12335

**Published:** 2021-06-28

**Authors:** Hiroya Mizusawa, Yuji Mimura, Haruhiko Utazu, Toshitaka Maejima

**Affiliations:** ^1^ Department of Urology National Hospital Organization Shinshu Ueda Medical Center Ueda Nagano Japan; ^2^ Department of Pathology and Laboratory Medicine National Hospital Organization Shinshu Ueda Medical Center Ueda Nagano Japan

**Keywords:** bladder tumor, edema, malignant tumor, proteinuria, renal biopsy

## Abstract

**Introduction:**

Nephrotic syndrome secondary to malignant disease accounts for approximately 10% of cases of nephrotic syndrome in adults. However, urothelial carcinoma of the bladder is a rare cancer, with only four cases reported to date.

**Case presentation:**

A 76‐year‐old man presented with chief complaints of edema and anorexia. Laboratory examinations revealed hypoalbuminemia and marked proteinuria, and computed tomography demonstrated multiple bladder tumors. Transurethral resection of the bladder tumors was performed. The pathological diagnosis was urothelial carcinoma with muscular invasion. The patient underwent simple cystectomy and ileal conduit formation, and proteinuria disappeared after 4 weeks. However, urethral recurrence was noted, and he died 35 months after cystectomy.

**Conclusion:**

Five cases including ours were clinically reviewed. Nephrotic symptoms improved relatively rapidly after surgery in all cases. In contrast to the poor preoperative general condition, postoperative improvement can be expected, and surgical treatment should, therefore, be considered.

**Keynote massage**

Cystectomy was performed on a patient with nephrotic syndrome secondary to muscle‐invasive bladder cancer, and nephrotic symptoms disappeared rapidly. Previous reports demonstrated improvement in nephrotic symptoms after surgery for bladder tumors. Surgery should be considered whenever possible for patients with secondary nephrotic syndrome due to bladder tumors.

## Introduction

Approximately 10% of adult patients with nephrotic syndrome have malignant disease.^1^, ^2^, ^3^ However, nephrotic syndrome complicated by bladder cancer is rare, with only four cases reported to date.^4^, ^5^, ^6^, ^7^ We report a case of nephrotic syndrome secondary to muscle‐invasive bladder cancer. We also clinically compare our case with previously reported cases.

## Case presentation

**Fig. 1:**
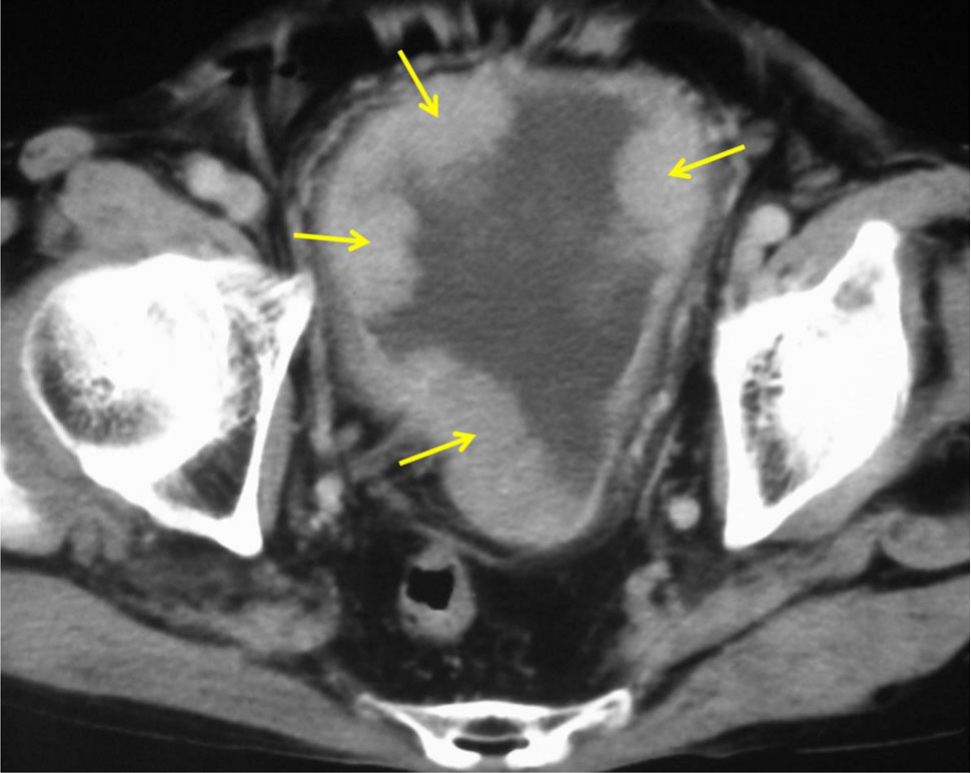
Thoracoabdominal contrast‐enhanced computed tomography. There were multiple large tumors in the bladder (arrows). There were no findings of lymph node enlargement or distant metastasis.

A 76‐year‐old man was referred to the gastroenterology department of our hospital by a local doctor for exacerbation of edema and anorexia. His medical history was noncontributory except for gastrectomy for a gastric ulcer. Blood biochemistry revealed anemia (red blood cell count: 304 × 10^6^/μL) and hypoproteinemia (serum total protein: 5.0 g/dL; albumin level: 1.8 g/dL), and urinalysis demonstrated proteinuria (2.9 g/day). His total cholesterol was 111 mg/dL. Multiple bladder tumors were suspected on thoracoabdominal contrast‐enhanced computed tomography and ultrasonography (Fig. 1), and the patient was referred to our department. There were no abnormal findings in either kidney on computed tomography. The patient noted occasional episodes of gross hematuria.

**Fig. 2:**
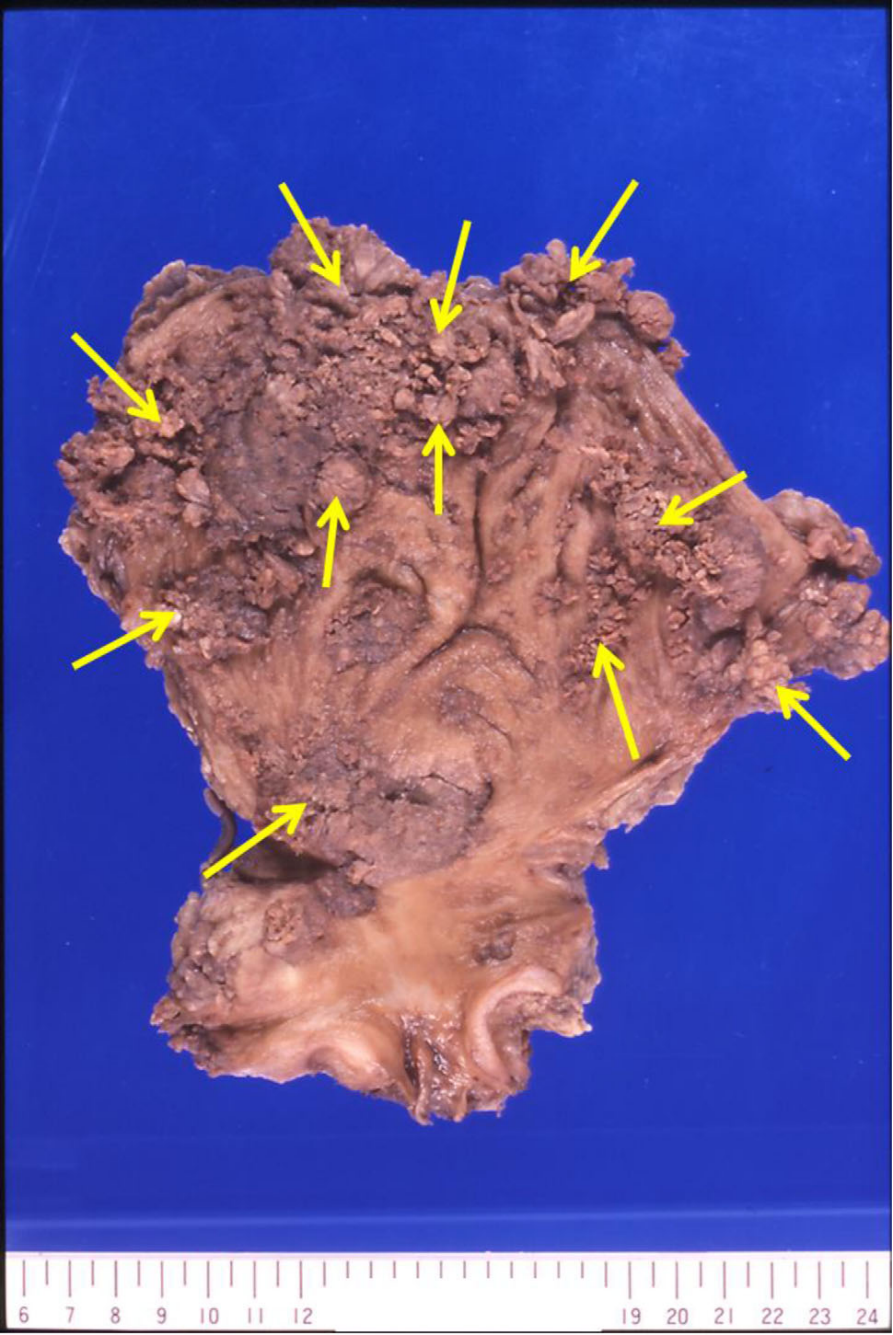
Macroscopic observation of the cystectomy specimen. Papillary tumors were widespread (arrows).

**Fig. 3:**
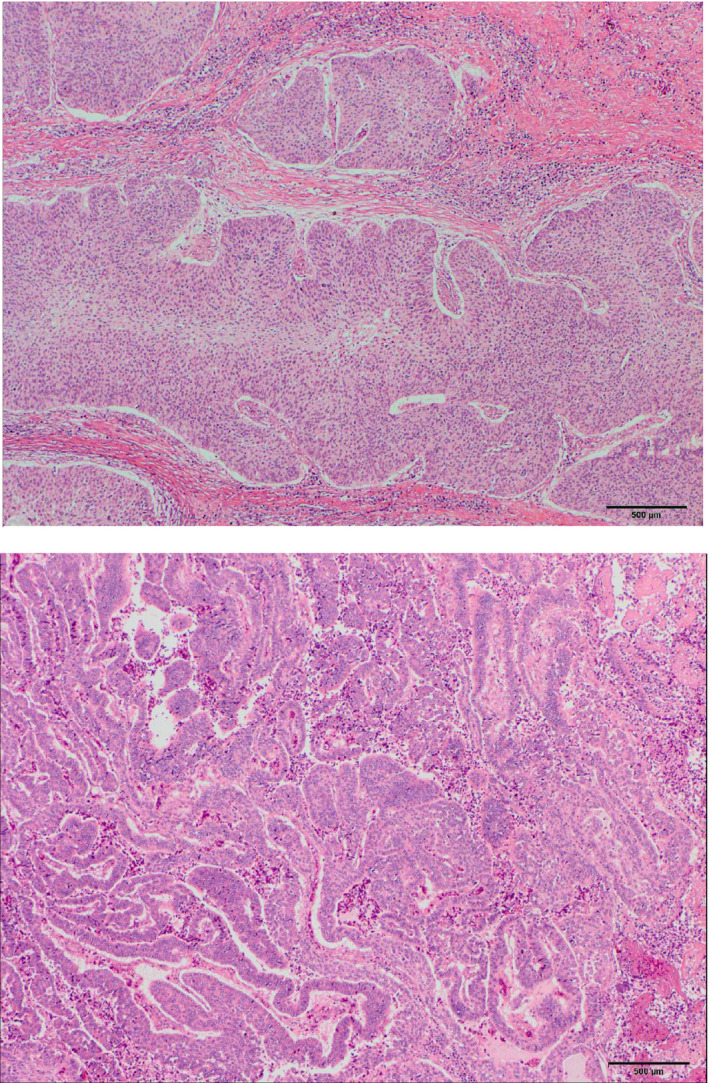
Microscopic observation of the cystectomy specimen. Urothelial carcinoma (upper) with glandular differentiation (lower). The bar length is 500 μm. Hematoxylin and eosin staining.

His height and weight were 150 cm and 42 kg, respectively. His blood pressure was 114/72 mmHg, pulse was 93 beats per minute, and body temperature was 36.0°C. The entire body was edematous, especially the face and legs. Cystoscopy revealed multiple sessile papillary tumors extending from both lateral walls to the posterior wall of the bladder. Urine cytology was class V. Transurethral resection of the bladder tumor was performed. The pathological diagnosis was urothelial carcinoma with glandular differentiation and invasion to the muscular layer. Further investigation demonstrated no evidence of metastasis. Edema gradually exacerbated, and his 24‐hour urine protein level increased to 11.1 g/day. The patient was diagnosed with nephrotic syndrome. Simple cystectomy and ileal conduit formation were performed. No typical lymph node dissection, urethrectomy, or renal biopsy was performed considering the patient’s general condition. Simple cystectomy was selected considering the anatomical condition of the prostate and general condition. Macroscopically, the bladder was almost filled with tumors (Fig. 2). Microscopically, lesions differentiated into glandular epithelia were extensive, and the following diagnosis was made: urothelial carcinoma with glandular differentiation, pT3a, u‐rt0, u‐lt0, ur0, ew0, ly1, v1, stage III (Fig. 3). The postoperative course was uneventful, and edema and proteinuria disappeared 4 weeks after surgery. His abnormally low blood levels of protein and albumin returned to normal 2 and 4 months after surgery, respectively. Adjuvant chemotherapy was not performed because he did not want to be admitted to the hospital. Urethral recurrence developed 20 months after cystectomy. There was no edema, with serum total protein and albumin levels of 6.2 g/dL and 3.0 g/dL, respectively. Urethrectomy was performed by using a perineal approach. Prostatectomy was not performed. The pathological diagnosis was urothelial carcinoma with glandular differentiation. Tumor invasion into the corpus spongiosum of the urethra was observed. As the patient did not wish to receive further treatment, he was treated palliatively and died 15 months after urethrectomy.

## Discussion

Approximately 10% of adult patients with nephrotic syndrome have malignant disease, with gastrointestinal and respiratory carcinomas being common.^1^, ^2^, ^3^

Our patient had nephrotic syndrome secondary to bladder cancer. Nephrotic syndrome associated with urothelial carcinoma of the bladder is a rare condition, with only four cases reported to date.^4^, ^5^, ^6^, ^7^ The clinical characteristics of urothelial carcinoma related to nephrotic syndrome remain unclear. These four previously reported cases together with ours are shown in Table 1. Regarding clinical symptoms, edema was the chief complaint in all cases except one. Eagan et al.^8^ clinically investigated 171 patients with nephrotic syndrome whose underlying disease was neoplasia, and they reported that symptoms of nephrotic syndrome preceded the diagnosis of malignant tumors in 73% of patients. Similar trends are likely in cases of nephrotic syndrome due to bladder cancer. Bladder tumors varied in size and had no specific trend. There were no patients with distant metastases and only one patient with a muscle‐invasive tumor. Thus, there may be no relationship between tumor‐related nephrotic syndrome and tumor stage in patients with bladder urothelial tumors.

**Table 1:** Clinical characteristics of reported cases of nephrotic syndrome secondary to urothelial carcinoma of the bladder

	*n* = 5
Age	66 (48‐78)
Men	4/5
Symptom	
Edema	4/5
Hematuria	2/5
Hydronephrosis	0/4
Creatinine (mg/dl)	
<1.0	2/5
≥1.0, <2.0	2/5
≥2.0	1/5
Tumor size (cm)	
<2	1/4
≥2, <5	1/4
≥5	2/4
Tumor depth	
<T2	2/3
≥T2	1/3
Treatment	
Cystectomy	3/5
TUR alone	2/5
Postoperative proteinuria	
Disappearance	3/5
Improvement	2/5
Renal biopsy	
MC	2/4
MPGN	1/4
MN	1/4

All patients underwent surgery. Regardless of the operative procedure or tumor size, proteinuria improved within 6 weeks after surgery in all patients. These results are inconsistent with those of Eagan et al.,^8^ who reported that nephrotic syndrome improved in only a limited number of patients despite treatment of their underlying disease. It should be noted, however, that the difficulties in past cancer treatment may have been responsible for the poor prognosis. Lefaucheur et al.^9^ reported a strong correlation between clinical remission of underlying malignancy and improvement of proteinuria. Indeed, nephrotic symptoms improved postoperatively in 88% of patients with esophageal cancer,^10^ in 78% of patients with gastric cancer,^11^ and in 100% of patients with rectal cancer,^12^ although the numbers were small.

This suggests that improvement in nephrotic symptoms is highly likely after resection of the primary lesion in patients with gastrointestinal cancer, and a similar trend was present for urothelial carcinoma, as described above.

Renal biopsy was performed in four cases, but not in ours, and there was no specific trend in histological findings, with minimal change disease, membranous nephropathy, and membranoproliferative glomerulonephritis all present. In patients with nephrotic syndrome secondary to malignant tumors, membranous nephropathy is generally associated with solid tumors, whereas minimal change disease is associated with nonsolid tumors such as Hodgkin’s disease. However, many exceptions to this generalization have been reported.^2^

Due to the severe symptoms of nephrotic syndrome, our patient had a poor general condition at the time of cystectomy. In addition to our patient, one patient was judged as unsuitable for surgery due to his/her poor general condition.^4^ However, surgical treatment should be considered for patients with symptoms of nephrotic syndrome because improvement of these symptoms can be expected postoperatively.

## Conclusion

We reported a patient with muscle‐invasive bladder cancer presenting with secondary nephrotic syndrome. Previous reports suggested that surgical treatment of bladder cancer can ameliorate nephrotic syndrome. For muscle‐invasive bladder cancer, surgery should be considered in cooperation with other departments regardless of the poor general condition of the patient.

## Conflict of interest

The authors declare no conflicts of interest.

## Approval of the research protocol by an institutional reviewer board

Not applicable.

## Informed consent

Not applicable.

## Registry and the registration no. of the study/trial

Not applicable.
